# Diversity of Microbial Eukaryotes Along the West Antarctic Peninsula in Austral Spring

**DOI:** 10.3389/fmicb.2022.844856

**Published:** 2022-05-16

**Authors:** Jean-David Grattepanche, Wade H. Jeffrey, Rebecca J. Gast, Robert W. Sanders

**Affiliations:** ^1^Department of Biology, Temple University, Philadelphia, PA, United States; ^2^Center for Environmental Diagnostics and Bioremediation, University of West Florida, Pensacola, FL, United States; ^3^Department of Biology, Woods Hole Oceanographic Institution, Pensacola, MA, United States

**Keywords:** picoplankton, nanoplankton, microplankton, Antarctic protists, high-throughput sequencing, RNA community

## Abstract

During a cruise from October to November 2019, along the West Antarctic Peninsula, between 64.32 and 68.37°S, we assessed the diversity and composition of the active microbial eukaryotic community within three size fractions: micro- (> 20 μm), nano- (20–5 μm), and pico-size fractions (5–0.2 μm). The communities and the environmental parameters displayed latitudinal gradients, and we observed a strong similarity in the microbial eukaryotic communities as well as the environmental parameters between the sub-surface and the deep chlorophyll maximum (DCM) depths. Chlorophyll concentrations were low, and the mixed layer was shallow for most of the 17 stations sampled. The richness of the microplankton was higher in Marguerite Bay (our southernmost stations), compared to more northern stations, while the diversity for the nano- and pico-plankton was relatively stable across latitude. The microplankton communities were dominated by autotrophs, mostly diatoms, while mixotrophs (phototrophs-consuming bacteria and kleptoplastidic ciliates, mostly alveolates, and cryptophytes) were the most abundant and active members of the nano- and picoplankton communities. While phototrophy was the dominant trophic mode, heterotrophy (mixotrophy, phagotrophy, and parasitism) tended to increase southward. The samples from Marguerite Bay showed a distinct community with a high diversity of nanoplankton predators, including spirotrich ciliates, and dinoflagellates, while cryptophytes were observed elsewhere. Some lineages were significantly related—either positively or negatively—to ice coverage (e.g., positive for Pelagophyceae, negative for Spirotrichea) and temperature (e.g., positive for Cryptophyceae, negative for Spirotrichea). This suggests that climate changes will have a strong impact on the microbial eukaryotic community.

## Introduction

The Southern Ocean, although connected to the other oceans, is separated from them by the polar front, which makes it a unique environment with many endemic species (Orsi et al., 1995). During the recent decades, the ice cover of Antarctica has changed dramatically due to climate change (Martinson et al., 2008; Comiso et al., 2017; Schofield et al., 2018; Clem et al., 2020), with a total increase of 3°C since 1951 and an anthropogenic impact that is not yet clear (Whitehouse et al., 2008; Ducklow et al., 2012; Turner et al., 2016). These changes in ice cover resulted in a release of nutrients and modification of the salinity in the area (Moline et al., 2004), potentially impacting the Antarctic ecosystem, as well as influencing the global climate (Cavanagh et al., 2021; Lin et al., 2021). Within the Southern Ocean, the West Antarctic Peninsula (WAP) is in itself unique, as three circumpolar Antarctic marine ecosystems: the Permanently Open Ocean Zone, the Seasonal Ice Zone, and the Coastal and Continental Shelf Zone, converge (Tréguer and Jacques, 1992) and result in strong environmental gradients that may affect diversity.

Microbial eukaryotes (protists) have a pivotal role in aquatic food webs and, in the Southern Ocean, are an important link between nutrients, bacteria, and higher trophic levels, including krill, penguins, and whales (Hempel, 1985; Hopkins, 1985; Murphy et al., 2007; Saba et al., 2014). Moreover, they can be heterotrophs (nano- and microzooplankton), phototrophs (phytoplankton), and mixotrophs/“mixoplankton” (combining phagotrophy and phototrophy; Stoecker et al., 2016; Leles et al., 2018; Ward, 2019). While an increasing number of studies have looked at the composition of the protists in marine systems, some areas are still notably understudied, including the polar regions (de Vargas et al., 2015; Pernice et al., 2015; Lin et al., 2021).

Over the past several decades, the large-scale sequencing of ribosomal RNA genes from environmental samples has indicated that the diversity of microbial taxa is much higher than expected, with a large number of species forming what has been called the rare biosphere (Sogin et al., 2006). The rare biosphere represents a large diversity of lineages present in the system, which are mostly at low abundance. Many of the sequences observed by DNA and/or RNA sequences have been assigned to lineages such as the SAR clade (Stramenopila, Aveolata, and Rhizaria), which dominates the species richness in the oceans, but a fair number of sequences are still taxonomically unidentified because lineages are not characterized or present in any molecular database (de Vargas et al., 2015; Massana et al., 2015; Grattepanche et al., 2018).

The Antarctic Peninsula region has been significantly impacted by global climate change. In addition to warming ocean temperatures, the seasonal sea ice period has decreased by as much as 3 months (Vaughan et al., 2003; Martinson et al., 2008; Stammerjohn et al., 2012). The phytoplankton community shifts in this region have been attributed to effects of climate change (Montes-Hugo et al., 2009; Brown et al., 2019). Traditionally, diatoms dominated coastal Antarctic Peninsula phytoplankton communities (Schofield et al., 2017), but they are being replaced by a community composed of smaller phytoplankton, particularly cryptophytes (nanophytoplankton) and *Micromonas* (picophytoplankton; Irion et al., 2020; Brown et al., 2021; Trefault et al., 2021). Some cryptophytes and *Micromonas polaris* are phototrophic plankton that consume bacteria, possibly to supplement some lack of macro- and micronutrients (Gonzalez et al., 1993; McKie-Krisberg and Sanders, 2014; Anderson et al., 2018). These changes in the phytoplankton community will not only affect the nutrients available for competing phytoplankters but also for the rest of the food web, including krill and penguins that rely on the phytoplankton directly or indirectly as a food source (Ducklow et al., 2007; Montes-Hugo et al., 2009; Schofield et al., 2010).

Here, we assessed the composition of the active microbial eukaryotic community by targeting ribosomal RNA, with an emphasis on three size fractions—the pico (0.2–5 μm), the nano (5–20 μm), and the micro (>20 μm)—along the Antarctic peninsula as a baseline to understanding continuing climate change. We hypothesized that (1) the SAR lineage (Stramenopiles, Alveolata, and Rhizaria) would be the dominant lineage in the Southern Ocean in terms of diversity and activity, as elsewhere; (2) the community would show a distribution related to the latitude as already observed for some ciliate lineages (tintinnids; Dolan et al., 2016); and, (3) during our sampling season (late austral spring and early summer), the diatoms would be the dominant phytoplankter.

## Materials and Methods

**FIGURE 1:**
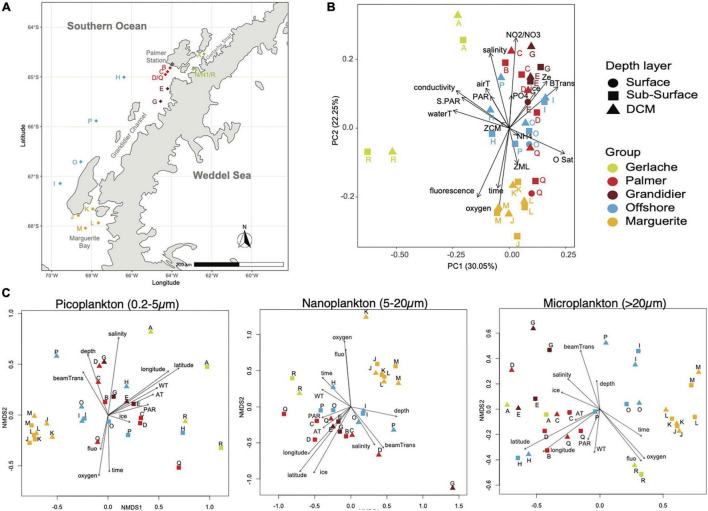
Sample distribution: **(A)** A chart of stations sampled during the NBP19-10 cruise. **(B)** Principal component analysis of environmental parameters showing five groups of samples related to geographical location. **(C)** Non-metric multidimensional scaling analysis of micro-, nano-, and pico-plankton, showing a similar pattern as the environmental parameter PCA.

During a cruise on the Nathaniel B. Palmer from November 6, 2019 to December 9, 2019 (NBP19-10), water samples were collected at the sub-surface (∼1-m depth) and at the deep chlorophyll maximum (DCM, ranging between 14- and 56-m depth) for 17 stations along the Antarctic peninsula (–64.54°; –62.37° to –68.05°; –68.30°; Figure 1A) using 12-L Niskin bottles mounted on a CTD rosette. At 3 stations, the surface was sampled with repeated bucket casts. We used bucket samples to find if there were distinct neustonic taxa that differed from the underlying water. Note that some stations were sampled at both the beginning and at the end of the cruise, so impact of time and geographical locations can be deciphered in our analyses.

### Environmental Parameters

Conductivity, water temperature, depth, salinity, oxygen, fluorescence, beam transmission, irradiance (PAR), oxygen saturation, time, latitude, and longitude measurements were collected at all casts with a CTD Sea-Bird SBE 9–11 plus V5.1 g, with WET Labs ECO-AFL/FL and C-Star probes. For quantification of chlorophyll a, triplicate samples of 100–250 ml were filtered onto a 25-mm GF/F filter (Whatman, United Kingdom) and frozen at –80°C until extraction. Filters were later extracted in 90% acetone overnight at –20°C, and fluorescence was determined with a Model TD-700 fluorometer (Turner Designs, Sunnyvale, CA, United States). Ice coverage was estimated by the N. B. Palmer crew based on direct observations during the CTD casts; northern stations had free-floating ice floes, while southern stations had pack ice.

Dissolved inorganic nutrient analyses were performed on the filtrates from GF/F filters using colorimetric methods with an Autoanalyzer. For each dissolved organic nutrient, a standard curve was calculated to determine the concentration of the nutrients in each sample. Dissolved inorganic phosphate (DIP) was analyzed as in Parsons et al. (1984) using a composite reagent, containing molybdic acid, ascorbic acid, and trivalent antimony, turning blue in the presence of DIP. For the quantification of NH_4_^+^, the method described in Holmes et al. (1999), which uses orthophthaldialdehyde (OPA) fluorescing in the presence of ammonium, was used. The concentration of NO^3–^ + NO^2–^ was determined using an acidic vanadium-(III)-solution and quantified by Griess–Ilosvay reaction, resulting in a purplish solution (Schnetger and Lehners, 2014). The sum of NH_4_^+^, NO^3–^, and NO^2–^ is also reported as dissolved inorganic nitrogen (DIN).

Bacterial production was performed on water samples using ^3^H-leucine (Smith and Azam, 1992). ^3^H-leucine (120 Ci mmol^–1^, Moravec Inc., Brea, CA) was added to six 1-ml samples in microfuge tubes to a final concentration of 10 nM. One sample was immediately killed with trichloroacetic acid (TCA) and served as the killed control. The samples were immediately capped, gently mixed, and incubated in the dark at *in situ* temperatures for 4 h. Incubations were terminated by the addition of TCA to a final concentration of 5%. The samples were processed using the microcentrifuge tube method of Smith and Azam (1992) and incorporation determined by liquid scintillation counting. Phytoplankton production was determined in 24-h incubations conducted at *in situ* temperature in deck incubators on the ship under 50% neutral density screens (Matrai et al., 1995). Six 16-ml seawater samples were placed in polyethylene bags (WhirlPak). ^14^C-Bicarbonate (Moravec Inc., Brea, CA) was added to a final concentration of 2.0 μCi ml^–1^. Three samples were then placed in opaque black plastic bags. The remaining three samples were placed inside a 50% neutral density screen envelope and all samples placed in a flowing seawater bath on the deck of the ship for 24 h. The samples were then retrieved, filtered onto 25-mm diameter GF/F filters, which were then rinsed with 10 ml of filtered seawater. The filters were then placed in liquid scintillation vials and acidified overnight after the addition of 100 μl of 20% HCl. Liquid scintillation cocktail (Ecolume) was added to each vial and fixed carbon determined by liquid scintillation counting.

### Nucleic Acid Samples

For each layer (surface and DCM), 20 L of seawater was serially filtered through a 20-μm nylon mesh and a 47-mm diameter/5-μm porosity nitrocellulose filter, and then 3 L of this < 5-μm seawater was filtered on a 47-mm diameter/porosity of 0.22-μm nitrocellulose filters. This resulted in three size fractions: > 20 μm referred as microplankton; 5–20 μm as nanoplankton and 0.22–5 μm as picoplankton for simplifying the notation (the classic limit between nano- and picoplankton is set at 3 μm). The filters were transferred to 1.5-ml centrifuge tubes and stored at –80°C until extraction.

While both DNA and RNA were simultaneously extracted and purified using an all prep DNA/RNA kit (Qiagen, Germany), only RNA was used in this study. We decided to use RNA to assess the plankton community because (1) DNA can be misleading as cysts, and dead cells can still exhibit DNA, and there is the potential presence of eDNA; (2) RNA reveals the most active organisms and, potentially, the ones having the biggest ecological impact. The use of RNA in the present study has the advantage of avoiding the contamination by dead cells and other eDNA particles. The presence of rRNA is also related to the metabolic activity of the community. RNA and DNA can both, therefore, be problematic when attempting to relate RNA- or DNA-based marker genes with abundance of cells. By utilizing RNA, this study focuses primarily on the active members of the community. Other possible biases of RNA include faster degradation of RNA compared to DNA (Cristescu, 2019) and errors introduced during reverse transcription. Some studies showed that (1) diversity estimates using SSU rRNA tend to be lower than diversity estimate using the SSU rRNA gene (i.e., DNA; Lanzén et al., 2011), (2) DNA estimates can miss some taxa detected only by RNA (Baldrian et al., 2012), and (3) there is high similarity between rDNA and rRNA in terms of number of OTUs (Cordier et al., 2022) but with some variation in read numbers (Rachik et al., 2018). This study, as others that have defined community composition based solely on molecular molecules (DNA or RNA), should be confirmed using an integrative approach, combining microscopy and/or physiology to better understand the ecology of microbial eukaryotes (Caron and Hu, 2019; Keeling, 2019).

A portion of the RNA was reverse transcribed into cDNA using Superscript III and random hexamers (Invitrogen, CA, United States). Amplicon generation used the cDNA samples, the Q5 polymerase (New England Biolabs, MA, United States), and primers targeting the hypervariable region V4 of the eukaryotic small subunit ribosomal gene (TAReuk454FWD1: 5′-CCA GCA SCY GCG GTA ATT CC-3′, TAReukREV3: 5′-ACT TTC GTT CTT GAT YRA-3′; Stoeck et al., 2010) with adaptors for Illumina sequencing requested by the University of Rhode Island genomics and sequencing center in order to perform Illumina MiSeq sequencing (2 × 300 cycles). The biases of this primer set are described and discussed in Hugerth et al. (2014), Piredda et al. (2017), and Clarke et al. (2021), which include difficulty in amplifying some taxonomic groups such as Amoebozoa and Foraminifera, and the current length limit of Illumina sequencing technology. However, each primer set will have some bias, for example, based on an *in silico* test using the Silva dataset, the V4 primers modified from Piredda et al. (2017) tend to be biased against Haptophyta, Excavata, Discoba, Cercozoa, and Retaria. We decided to use this primer set as the V4 region is more taxonomically informative than other hypervariable regions (Hugerth et al., 2014), and we used the original primer set described by Stoeck et al. (2010) in order to be able to compare our data to published works (Massana et al., 2015; Swalethorp et al., 2019; Giner et al., 2020). To reduce the bias due to amplification, each PCR was carried out in triplicate and pooled before being sent to the sequencing center (Lahr and Katz, 2009).

### Bioinformatics

To analyze the amplicon data, two datasets were considered: the paired-end read (P-E) and the forward read (FWD) datasets. The first is more sensible for assigning taxonomy as DNA fragments are longer but is more subject to issues related to the pair-end process (reverse reads of poor quality, an overlapping region that is too short, variable fragment size). The forward read dataset alone could possibly access a more “complete” community. The datasets were analyzed as previously described (Sisson et al., 2018; Grattepanche et al., 2019). In summary, paired-end reads were merged using BBMerge (Bushnell et al., 2017), and then both datasets were dereplicated using Vsearch (Rognes et al., 2016). Reads with unknown characters (N) were discarded, and OTUs were picked using SWARM2 and a distance of 1 (Mahe et al., 2015). We compared the diversity assessment using our current method, which involved SWARM to a pipeline using DADA2 (Callahan et al., 2016) in a previous paper (Grattepanche and Katz, 2020). In summary, both approaches were similar in the diversity assessment. However, we observed that some reads were discarded by DADA2 and considered as noise, while many others were kept (overestimation of diversity). Another big difference is that DADA2 analyzes each sample independently, while SWARM analyzes all the samples at once, which allows consideration as outliers if they appear in multiple samples. We decided to use SWARM to better control the OTU library, which includes correction of PCR and sequencing errors. Chimeras were identified using Uchime-*de novo* (Edgar et al., 2011) implemented in Vsearch and removed; singletons (OTU with only 1 read) and contaminants were discarded using local alignment water implemented in EMBOSS (Rice et al., 2000) and a cutoff similarity of 50%. The resulting sequence file was aligned against a guided alignment of PR2 data (Guillou et al., 2012). Phylogenic trees were built to identify non-eukaryotic OTUs for removal using RAxML-EPA (Stamatakis, 2014; Barbera et al., 2019). These phylogenies and a similarity approach (assignment by similarity using usearch_global implemented in Vsearch) were used to assign the taxonomy to each OTU, including at least 3 taxonomic ranks (e.g., SAR, Stramenopila, Ochrophyta or Opisthokonta, Choanoflagellata, Acanthoecida). Alignments are visually inspected in case of disagreement between the two methods of taxonomic assignment.

### Statistics

Principal component analysis on the environmental data was performed to identify distribution patterns (by a depth layer, size, geographical location, related to nutrients, etc.), and the data interpolating empirical orthogonal function were used to extrapolate missing data (Beckers and Rixen, 2003).

To compare the community composition across our samples from different size fractions, stations, and depth layers, each sample was rarefied at 25,000 reads for the paired-end read (P-E) dataset. The same analyses were performed for the forward read (FWD) dataset, but without rarefaction to avoid some bias (e.g., introduction of artificial variation) and using proportions instead of number of reads (McMurdie and Holmes, 2014).

Non-metric multidimensional scaling (NMDS) with the Fast Unifrac dissimilarity index (Hamady et al., 2009) was used to assess the similarity between samples with the phyloseq and phyloseqCompanion packages (McMurdie and Holmes, 2013; Stagaman, 2019) implemented in R (R Core Team, 2021). To confirm the community group observed, ANOVA was performed using adonis2 implemented in the vegan package (Oksanen et al., 2020) from R, in which the data were randomly permuted 999 times, and the best model selected based on lowest Akaike information criterion corrected. The function envfit implemented in the vegan package was used to relate the axis of our ordination to environmental factors.

## Results

### Environmental Pattern

The stations sampled varied in depth, ranging from 135 to 1,400 m, with the shallower depths near shore across the latitudinal range. The salinity, temperature, and oxygen ranged from 33.17 to 34.20 PSU, 1.8–0.9°C, and 5.9–7.9 ml L^–1^, respectively. The chlorophyll a concentration ranged from 0.1 to 3.25 mg m^–3^ (Table 1). The depth of the mixed layer (ZML) and the depth of the deep chlorophyll maximum (ZCM) showed a negative relationship with the latitude and longitude (Supplementary Figure 1A), indicating shallower mixed layer and deep chlorophyll maximum depths in the northern stations, compared to the southern (Table 1). Overall, temperatures (air and water), ice coverage (% of floating ice observed at the sea surface), conductivity, and nutrients decreased significantly going southward (Table 1 and Supplementary Figure 1A).

**TABLE 1:** Mean (standard deviation) of environmental parameters observed during the austral spring 2019 in different regions of the WAP.

						DCM					Surface				
	Gerlache	Palmer	Grandidier	Offshore	Marguerite	Gerlache	Palmer	Grandidier	Offshore	Marguerite	Gerlache	Palmer	Grandidier	Offshore	Marguerite
ZML (m)	18.81 ± 4.93	13.12 ± 1.73	16.24 ± 2.17	30.58 ± 18.81	35.64 ± 5.75										
Ze (m)	39.94 ± 9.39	52.1 ± 5.52	63.16 ± 6.51	57.2 ± 14.84	43.56 ± 4.91										
ZCM (m)	16.5 ± 11.07	17.2 ± 15.32	7.72 ± 1.08	28.38 ± 11.82	23.51 ± 16.25										
Bottom (m)	437.67 ± 234.24	1,156 ± 343.05	175 ± 0	541.11 ± 100.46	427.25 ± 253.8										
Ice (%)	3.33 ± 5.16	92.5 ± 4.63	70 ± 0	42.22 ± 44.1	2.5 ± 4.63										
Air T (°C)	0.6 ± 0.91	–1.44 ± 0.11	–1.32 ± 0.16	–1.39 ± 0.17	–3.05 ± 3.12										
Surf_PAR	716.79 ± 529.61	352.15 ± 255.08	108.22 ± 25.33	451.46 ± 291.97	219.41 ± 124.77										
Depth (m)	11.88 ± 17.33	14.48 ± 17.59	10.5 ± 14.78	15.84 ± 20	18.93 ± 19.01	22.77 ± 19.88	32.01 ± 16.46	24.75 ± 14	33.91 ± 16.81	35.89 ± 8.72	0.99 ± 0	3.96 ± 6.1	0.99 ± 0	1.39 ± 0.54	1.98 ± 0.81
Watert (°C)	0.14 ± 0.57	–1.59 ± 0.16	–1.46 ± 0.11	–1.63 ± 0.22	–1.59 ± 0.12	0.03 ± 0.38	–1.51 ± 0.15	–1.42 ± 0.03	–1.58 ± 0.27	–1.57 ± 0.14	0.26 ± 0.79	–1.64 ± 0.16	–1.49 ± 0.14	–1.67 ± 0.2	–1.62 ± 0.11
Conductivity (mS cm^–1^)	28.43 ± 0.43	26.68 ± 0.32	26.88 ± 0.1	26.81 ± 0.24	26.63 ± 0.13	28.4 ± 0.29	26.86 ± 0.28	26.96 ± 0.12	26.93 ± 0.24	26.67 ± 0.15	28.47 ± 0.61	26.57 ± 0.32	26.83 ± 0.07	26.71 ± 0.2	26.58 ± 0.11
Salinity (PSU)	34.03 ± 0.16	33.6 ± 0.32	33.73 ± 0.12	33.83 ± 0.14	33.53 ± 0.04	34.1 ± 0.17	33.75 ± 0.29	33.79 ± 0.18	33.92 ± 0.06	33.54 ± 0.04	33.95 ± 0.15	33.51 ± 0.33	33.7 ± 0.07	33.75 ± 0.15	33.51 ± 0.04
PAR	252.79 ± 393.62	213.09 ± 322.5	34.2 ± 25.94	67.35 ± 62.8	30.97 ± 31.8	18.15 ± 14.21	17.84 ± 17.95	7.17 ± 4.37	7.67 ± 8.23	2.27 ± 2.69	487.42 ± 471.15	330.23 ± 368.93	52.21 ± 10.92	115.09 ± 37.77	59.66 ± 12.52
BeamTrans (%)	92.37 ± 5.59	97.96 ± 1.44	98.75 ± 0.26	98.27 ± 0.91	96.58 ± 0.86	93.02 ± 5.29	98.54 ± 1.1	98.83 ± 0.36	98.57 ± 0.76	96.67 ± 0.86	91.72 ± 6.99	97.61 ± 1.62	98.69 ± 0.24	98.04 ± 1.04	96.5 ± 0.98
NH_4_^+^ (μM)	1.26 ± 0.07	1.44 ± 0.06	1.29 ± 0.05	1.28 ± 0.07	1.29 ± 0.01	1.25 ± 0.04	1.4 ± 0.07	1.27 ± 0.06	1.25 ± 0.04	1.29 ± 0.01	1.27 ± 0.1	1.47 ± 0.01	1.32 ± 0.02	1.31 ± 0.09	1.29 ± 0.02
NO_2_^–^/NO_3_^–^ (μM)	29.32 ± 1.67	29.75 ± 1.71	30.55 ± 0.3	29.1 ± 0.83	26.08 ± 0.73	29.85 ± 1.08	30.24 ± 1.64	30.6 ± 0.13	29.44 ± 0.85	26.49 ± 0.9	28.78 ± 2.22	29.26 ± 2.27	30.51 ± 0.5	28.77 ± 0.76	25.68 ± 0.15
PO_4_^3–^ (μM)	3.12 ± 0.15	3.18 ± 0.15	3.13 ± 0.13	3.12 ± 0.18	2.93 ± 0.17	3.17 ± 0.11	3.19 ± 0.16	3.21 ± 0.06	3.24 ± 0.14	2.96 ± 0.09	3.06 ± 0.18	3.17 ± 0.19	3.05 ± 0.16	3 ± 0.11	2.9 ± 0.24
oxygen (mL L^–1^)	6.87 ± 0.65	7.07 ± 0.53	6.46 ± 0.06	7.31 ± 0.36	7.71 ± 0.18	6.63 ± 0.69	7.07 ± 0.28	6.4 ± 0.05	7.2 ± 0.43	7.63 ± 0.17	7.12 ± 0.62	7.07 ± 0.68	6.5 ± 0.02	7.4 ± 0.32	7.79 ± 0.17
Oxygen Saturation (mL L^–1^)	8.04 ± 0.11	8.44 ± 0.05	8.4 ± 0.02	8.43 ± 0.05	8.44 ± 0.03	8.05 ± 0.08	8.41 ± 0.04	8.39 ± 0.01	8.42 ± 0.06	8.44 ± 0.03	8.02 ± 0.16	8.45 ± 0.05	8.41 ± 0.03	8.45 ± 0.05	8.45 ± 0.03
Fluorescence (mg m^–3^)	1.65 ± 1.25	0.62 ± 0.45	0.33 ± 0.14	0.41 ± 0.24	1.68 ± 0.6	2.19 ± 1.51	0.6 ± 0.52	0.33 ± 0.21	0.47 ± 0.31	1.92 ± 0.63	1.11 ± 0.85	0.63 ± 0.46	0.34 ± 0.14	0.35 ± 0.18	1.43 ± 0.53
Chlorophyll a (mg m^–3^)	1.53 ± 1.02	0.73 ± 0.42	0.28 ± 0.13	0.35 ± 0.16	1.11 ± 0.23	1.29 ± 0.7	0.5 ± 0.39	0.22 ± 0.16	0.3 ± 0.12	1.05 ± 0.15	1.77 ± 1.39	0.89 ± 0.42	0.32 ± 0.11	0.38 ± 0.19	1.17 ± 0.31
Primary production (μgC L^–1^ d^–1^)	14.54 ± 0.54	4.8 ± 4.15	1.16 ± 0.69	2.07 ± 1.26	5.07 ± 1.31	2.1 ± 1.24	0.92 ± 0.8	2.14 ± 2	4.27 ± 1.1		6.6 ± 4.64	1.33 ± 0.74	2.01 ± 0.71	5.87 ± 1.05	
Bacteria (10^8^ L^–1^)	1.97 ± 0.6	1.68 ± 0.45	1.03 ± 0.16	2.08 ± 0.67	1.52 ± 0.46	2.11 ± 0.74	1.59 ± 0.05	1.17 ± 0.08	1.85 ± 0.91	1.53 ± 0.46	1.84 ± 0.67	1.75 ± 0.62	0.94 ± 0.14	2.24 ± 0.52	1.5 ± 0.53
Bacterial production (μgC L^–1^ d^–1^)	6.97 ± 3.64	7.2 ± 4.99	1.92 ± 1.04	3.36 ± 2.52	7.42 ± 1.78	6.85 ± 4.35	6.26 ± 8	1.44 ± 1.17	2.03 ± 1.53	6.98 ± 2.44	7.08 ± 3.76	7.76 ± 3.25	2.24 ± 1.05	4.43 ± 2.78	7.86 ± 0.96
Richness	964 ± 564	1,043 ± 94	1,179 ± 543	1,132 ± 147	937 ± 86	1,031 ± 602	1,061 ± 60	1,131 ± 233	1,062 ± 185	970 ± 111	897 ± 653	1,032 ± 116	1,227 ± 710	1,187 ± 95	905 ± 47
Chao1 index	1,536 ± 904	1,616 ± 195	1,945 ± 902	1,762 ± 226	1,462 ± 126	1,621 ± 980	1,636 ± 21	1,818 ± 402	1,721 ± 340	1,506 ± 120	1,450 ± 1,036	1,605 ± 256	2,071 ± 1,196	1,795 ± 111	1,418 ± 132
Shannon index	3.25 ± 1.78	3.56 ± 0.21	3.51 ± 1.59	3.74 ± 0.37	3.12 ± 0.24	3.47 ± 2	3.58 ± 0.29	3.46 ± 0.45	3.48 ± 0.3	3.13 ± 0.35	3.04 ± 1.95	3.56 ± 0.18	3.56 ± 2.06	3.96 ± 0.29	3.11 ± 0.1
pico.Richness	964 ± 564	1,043 ± 94	1,179 ± 543	1,132 ± 147	937 ± 86	1,031 ± 602	1,061 ± 60	1,131 ± 233	1,062 ± 185	970 ± 111	897 ± 653	1,032 ± 116	1,227 ± 710	1,187 ± 95	905 ± 47
nano.Richness	966 ± 499	1,090 ± 261	1,133 ± 676	1,381 ± 496	1,327 ± 157	1,004 ± 580	1,150 ± 366	921 ± 789	1,320 ± 142	1,311 ± 208	928 ± 536	1,054 ± 218	1,344 ± 777	1,442 ± 680	1,343 ± 117
micro.Richness	697 ± 395	703 ± 26	643 ± 342	1,075 ± 378	980 ± 132	606 ± 378	707 ± 9	572 ± 170	1,088 ± 377	966 ± 184	788 ± 485	701 ± 34	715 ± 462	1,064 ± 423	995 ± 77

*ZML = mixed layer depth, ZML_T estimated using temperature, ZML_TS using temperature and salinity, and ZML_TSP using temperature, salinity, and pressure. Ze = depth of the (eu)photic zone, ZCM = depth of chlorophyll maximum, bottom = maximum depth, time is Julian day,% ice is the estimated percentage of ice coverage, airT = air temperature, surfPAR = surface photo-active radiation, waterT = water temperature, beamTrans = beam transmission.*

To have a better understanding of how the environmental parameters were distributed across “habitats,” principal component analysis was performed using salinity, conductivity, water, and air temperature, dissolved oxygen, DO saturation, fluorescence, beam transmission, maximal depth, sampling depth, ice coverage, PAR and surface PAR, depths of the deep chlorophyll maximum of the euphotic zone and of the mixed layer (Figure 1B). The first two axes of the PCA explain more than 47% of the variability in our dataset. Overall, no clear distinction between the three depth layers sampled was observed: surface (sampled with a bucket), sub-surface (1 m, sampled with the CTD-rosette), and DCM samples, because the different depth layers for the same station tend to cluster close to each other (Figure 1B). Five groups were identified from the analysis of environmental parameters that related to their geographical location: a southern group located in Marguerite Bay (MB hereafter), a northern group located in Gerlache Strait (GS hereafter), two middle groups situated between Grandidier Channel (GC) and Anvers Island, where the USAP Palmer station is located (PS hereafter) and, lastly, an offshore group (OFF hereafter).

The GS group is composed of samples with higher conductivity and temperature, and, in some cases, a higher PAR. This group of samples represented a shallower mixed layer.

PS and GC groups are composed of stations with a deeper photic zone, a higher beam transmission, a lower fluorescence, and more extensive ice coverage. Lower bacterial abundance and productivity were observed for these groups (Table 1). The PS stations also showed the highest NH_4_^+^ concentration (Table 1).

The OFF group is composed of stations with higher oxygen saturation that tend to have a deeper mixed layer and a deeper chlorophyll maximum depth, while the PAR is lower (Table 1).

The MB stations also had higher oxygen concentrations and a deeper mixed layer. The MB stations also presented the lowest concentration of nitrite/nitrate and high concentration of chlorophyll (Table 1).

### Overall Community Composition

#### Richness, Alpha Diversity, and Reads Number

Overall, the eukaryotic community, assessed from 18S rRNA, was composed of 200,760 OTUs from almost 3.8 million reads in the dataset composed of OTUs with paired-end reads (P-E reads hereafter; Supplementary Dataset 1). Of these OTUs, nearly 64% were present in only one sample and accounted for less than 4% of the paired-end reads. Conversely, only 2 OTUs (i.e., 0.001% of the OTUs), which encompassed 10% of the paired-end reads (386,817 P-E reads), were observed in all the samples (location, depth, and size fractions). The second dataset, which is composed of only forward reads (FWD reads hereafter; Supplementary Dataset 2), was used to assess the bias related to length of the sequence and issues with paired-end assembling. This dataset is composed of 87,214 OTUs, for a total of almost 3.5 million FWD reads. Overall, this dataset showed the same trend with a large number of OTUs observed in only 1 sample (29,891 OTUs, i.e., 34% of the OTUs and 64,704 FWD reads, i.e., almost 2% of FWD reads), and 5 OTUs observed in all the samples, representing almost 15% of the FWD reads (525,160 FWD reads).

To look at the distribution within the samples, stations, depth layers, and size fractions, only OTUs with at least 100 reads are considered in our subsequent analyses (labeled as abundant OTUs hereafter; Supplementary Dataset 3), and the samples were rarefied at 25,000 reads, which represented 1,857 abundant and active OTUs for more than 2 million paired-end reads. Each sample was represented by an average of 574 abundant OTUs (from 333 to 943 OTUs). The surface samples had an average of 588 abundant OTUs (from 333 to 790; 38 samples), and the DCM samples had 558 OTUs (between 375 and 943; 39 samples). Only a few OTUs showed a clear relationship for a single depth layer (1 and 9 abundant OTUs representing 40 and 496 reads for surface and DCM layers, respectively). Even considering a cutoff (i.e., if 95% of the reads for the OTU are observed in one depth layer, then the OTUs are considered as specific to this depth layer only, the 5% representing potential bias in the filtration or in the molecular steps), this trend is persistent (4 and 24 abundant OTUs representing 298 and 8,896 reads for surface and DCM layers, respectively). This suggests that most taxa are not restricted to a sampling depth but are active across layers at a given station during our cruise.

#### Size Distribution

The diversity was then examined within each size fraction. The nano-size fractions tended to have higher richness (an average of 672 and 656 abundant OTUs for surface and DCM layers, respectively), while the pico-size fractions tended to show lower richness (an average of 530 and 519 abundant OTUs for surface and DCM layers, respectively; Supplementary Figure 2A). At least 12% of the abundant OTUs were specific to a size fraction and represented less than 3% of the reads (i.e., 10.2, 0.3, and 1.6% of the abundant OTUs were specific to the micro-, nano-, and pico-size fractions, respectively). Considering the same cutoff as before (i.e., 95% of the reads), almost 40% of the abundant OTUs for a third of the reads showed a clear size fraction signature, with 28, 5, and 6% of the abundant OTUs representing 27, 2, and 5% of the reads that were classified as specific to the micro-, nano-, and pico-size fractions, respectively. This trend is also consistent when grouping OTUs in morphospecies (based on assignment to known taxa; Supplementary Figure 2B). This suggests that the taxa do not tend to vary in their size distribution, especially for the micro-size fraction. It is interesting to note that only 14 OTUs (for a total of 1,857 abundant OTUs), representing 6,089 reads (out of more than 2 million reads), were present in both the micro- and pico-size fractions, suggesting that our filtration methods and a molecular approach were appropriate, and/or that size disjunction within microbial eukaryotes is rare.

#### Species Composition

**FIGURE 2:**
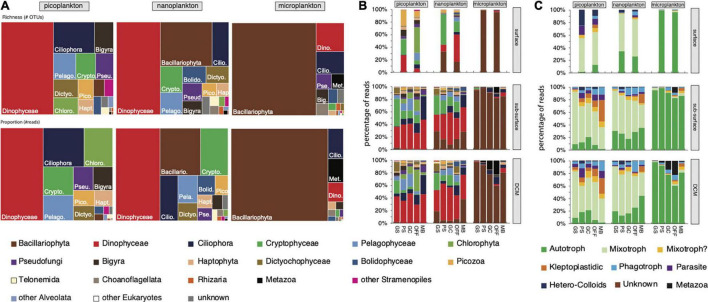
Distribution of **(A)** lineages per size fraction, and **(B)** lineages and **(C)** trophic mode by size fraction, depth and group, showing the distinct composition of micro-, nano-, and picoplankton and the pattern related to the geographical location. Surface and sub-surface samples showed a different distribution, while subsurface and DCM samples shared a more comparable composition. **(B,C)** Bar graphs used the abundant OTUs, while **(A)** the treemap graph used all P-E OTUs.

Overall, diatoms dominated the micro-size fractions, while dinoflagellates, ciliates, diatoms, and cryptophytes dominated the nano- and pico-size fractions assessed from 18S rRNA (Figure 2A and Supplementary Figure 3A). Other groups were also present but in lower proportions, and included Haptophyta, Chlorophyta, Opalozoa, Picozoa, Pseudofungi, Sagenista lineages, and the clade named Opisthokonta_X in the PR2 database (Guillou et al., 2012). Our OTUs showed similarity to 1,139 morphospecies reported in our database, with 20% of these taxa observed in at least 50% of the samples, and 14 taxa in all the samples (taxa can be represented by multiple OTUs). The SAR clade [Stramenopiles (representing 37% of the reads), Alveolata (31%) and Rhizaria (< 1%)] was the dominant group in our samples with 867 morphospecies (76% of the morphospecies) and 68% of the FWD reads. The diatoms had the most taxa identified with 296 taxa, followed by 276 dinoflagellate and 139 ciliate taxa. Within the dominant taxa, we observed *Corethron inerme* (Stramenopiles), *Heterocapsa rotundata* (Dinoflagellata), *Prorocentrum* sp. (Dinoflagellata), *Geminigera cryophila* (Cryptophyta), *Porosira pseudodenticulata* (Stramenopiles), *Pelagomona scalceolata* (Stramenopiles), *Strombidium caudispina* (Ciliophora), *Phaeocystis antarctica* (Haptophyta), and *Micromonas polaris* (Chlorophyta), each representing more than 1% of the total read number. Unsurprisingly, more than 20% of the reads were represented by uncharacterized OTUs (low similarity to known sequences in our database). No foraminifera were detected using our 18S rRNA primer set, while some were observed using a binocular microscope during the samplings.

### Community Pattern

#### Geographical Distribution

Overall, the diversity is relatively constant across latitude, with a slight increase in the samples collected in Marguerite Bay (MB), especially for the micro- and the nanoplankton (Supplementary Figure 2). Alpha diversity assessed by richness (number of OTUs), Shannon or Chao1 indices, using OTUs or morphospecies showed the same trend: higher alpha diversity for the samples collected in Marguerite Bay. This higher diversity for the MB group is particularly marked when using Shannon index, indicating a more diverse community, with an even distribution of abundance (as estimated by the number of reads). The alpha diversity estimated using OTU number and Chao1 index was generally stable for the surface (bucket), sub-surface, and DCM layers and for the three size fractions (Supplementary Figure 2A). This suggests that the total number of OTUs (including the missing ones as estimated by the Chao1 diversity index) for each sample is very similar. While the numbers tend to be lower when considering the morphospecies, we observed the same trend, i.e., higher diversity in the MB group, especially for the microplankton (Supplementary Figure 2B). The picoplankton diversity was relatively stable whatever the station or depth layer and tended to slightly decrease with time [from our first station (A) to our last station (R)]. As previously mentioned, the cruise track doubled back on itself so that some of the later stations were geographically close to the earliest stations.

The non-metric multidimensional scaling (NMDS) with the Fast Unifrac dissimilarity index showed that the microbial eukaryotic community, assessed from 18S rRNA, was strongly impacted by the environmental parameters (Figure 1C). However, the biggest factor differentiating the protist assemblage was size fraction. In fact, using permutational multivariate ANOVA of distance matrices (adonis), the size significantly explains 22% of the variance of our communities (Supplementary Table 1). Then, the size, in conjunction with the groups observed with our environmental parameters, explains another 12% of the variance, while the groups alone are responsible for 10% of the variance within our communities (Supplementary Table 1). So, almost 45% of the variance of our community is explained by the size fraction and its location (i.e., community groups match the environmental groups).

The NMDS and envfit analyses were performed independently for each size fraction. The distribution of the picoplankton was related to the location (longitude and latitude), the depth of sampling, salinity, oxygen concentration, and, to the date of sample collection (Figure 1C and Supplementary Table 1A). The nanoplankton distribution was significantly associated with the location (longitude and latitude), the percentage of ice coverage and with the oxygen concentration (Figure 1C and Supplementary Table 1A). The distribution of the microplankton was related to the location (longitude and latitude), oxygen concentration, fluorescence (proxy for the phytoplankton biomass), and to the beam transmission (proxy for the number of particles in the water column; Figure 1C and Supplementary Table 1A).

Some lineages were significantly related—either positively or negatively—to environmental parameters (Supplementary Table 1). Some lineages showed a latitudinal gradient such as the Cryptophyceae (decrease in proportion southward) and spirotrich and other ciliates (increase southward; Supplementary Figure 1B and Figure 2B). These relationships can also be related to the temperature (positive relationship between cryptophyte and water temperature, and, inversely, negative correlation between spirotrich ciliates and temperature) or ice coverage (negative correlation between spirotrich ciliates and ice coverage; Supplementary Figure 1B). Pelagophyceae showed a significant and positive relationship with ice coverage and nitrite/nitrate and tended to decrease in proportion southward (Supplementary Figure 1B).

#### Dominant Taxa

The community from Marguerite Bay (MB) shows the most distinctive assemblage compared to the other groups for the three size fractions. Overall, 28 abundant OTUs represented more than 57% of the reads (Dataset S3). These 28 abundant OTUs accounted for 25–84%, 41–69%, and 42–78% of the reads of the micro-, nano-, and pico-plankton, respectively. The microplanktonic OTUs related to *Pirsonia formosa* (Oomycetes), a parasitoid of diatoms, and to the diatom *Porosira pseudodenticulata* (Supplementary Figure 3B) that dominated the community in the MB group, while the other groups were dominated by OTUs related to the diatom *Corethron inerme*. A larger number of OTUs were related to Ciliophora, including the Oligotrich *Strombidium caudispina* and the tintinnid *Cymatocylis calcyformis* present in all size fractions but mostly in the nano- and pico-plankton (Supplementary Figure 3B). A large portion of the nano- and picoplankton was composed of dinoflagellates.

The spatial distribution of each OTU was considered to identify an OTU specific to a depth layer or to a group (i.e., at least 90% of its reads were observed in this depth layer or group). For this analysis, only abundant OTUs that occurred in at least 2 samples were considered, which reduced the number of OTUs from 1,857 to 1,856. Only a small fraction of the OTUs is specific to a depth layer. Only 1 OTU accounting for 395 reads was specific to the bucket samples, 2 and 35 OTUs were specific to the sub-surface and DCM samples, respectively (accounting for 186 and 15,580 reads, respectively). The most abundant DCM-specific OTUs are related to ciliates (e.g., OTUs related to *Leegardiella* and other Strombidiidae). We also observed 112 OTUs specific to a group, representing 56,417 reads (i.e., 5% of the OTUs for less than 3% of the reads). Half of these OTUs are the members of the Bacillariophyta (61 OTUs for 24,577 reads). Most of the group specific OTUs are from the MB area with 83 OTUs and 48,173 reads (75% of the area-specific OTUs and 85% of the area-specific reads), with 47 and 37% of the reads represented by Bacillariophyta and the parasite *Pirsonia* (Supplementary Figure 4).

#### Trophic Mode

The trophic mode of each abundant OTU was estimated based on the literature (Leles et al., 2017; Faure et al., 2019; see Supplementary Dataset 3 for additional references) to better understand the relationship between the geographical distribution and the community pattern (Figure 2C). Overall, the microplankton was mostly composed of phytoplankton, especially diatoms; a big proportion of nano- and pico- plankton corresponds to mixoplankton, including mixotrophic dinoflagellates, such as *Heterocapsa rotundata*, *Prorocentrum* sp., and *Warnowia* sp., cryptophytes (*Geminigera cryophila*) and *Micromonas polaris*. The contribution of phagotrophic (heterotrophic dinoflagellates such as *Gyrodinium* cf. *spirale; Leegardiella* sp. and other unknown ciliates) and kleptopladistidic (mostly oligotrich ciliates) lineages tends to increase going southward. Parasites were also present but had only a limited contribution (less than 4% of the total read number; Figure 2C). While the depth layers showed slightly different lineage proportions—more cryptophytes in the sub-surface layer compared to the DCM layer, more Dinophyceae and Mamiellophyceae (mostly *Micromonas polaris*) in the DCM layer, the trophic mode distribution was mostly similar between the sub-surface and the DCM depths. We noted the large contribution of picozoa (*Picomonas*), known as consumers of colloid particles, in the surface layer (collected with a bucket).

## Discussion

In the austral late spring and early summer, the microbial eukaryotic community along the western Antarctic peninsula was strongly influenced by environmental factors that presented a distribution related to the sampling location. Five groups were observed in all three size fractions — micro-, nano-, and pico-size fractions (Figure 1C). These groups were also examined against the environmental parameters, including temperature, salinity, PAR, oxygen, amount of particulates in the water column (beam transmission), and structure of the water column (depths of the mixed layer, the euphotic zone, and, to a lesser extent, to the deep chlorophyll maximum; Figure 1B). The most dissimilar community assessed using 18S rRNA, compared to the others, was observed in Marguerite Bay (MB), i.e., our southernmost sampling location. Conversely, the communities from the near shore stations in the Gerlache Strait (GS) and Grandidier Channel (GC) were phylogenetically most similar, especially for the pico- and nano-plankton and grouped together despite the differences in environmental parameters shown in the PCA (Figures 1B,C). The latitudinal gradient for the environmental factors and for the community composition corresponds also to a gradient in a trophic mode with an increase of heterotrophy (as we analyzed RNA, which combines both presence and activity), including both phagotrophy and mixotrophy, toward the south.

### Spring in the Western Antarctic Peninsula

Overall, much of the data on eukaryotic microorganisms, especially phytoplankton, for the WAP come from January or later in the year—in particular from cruises associated with the Palmer Station Long-Term Ecological Research program (Ducklow et al., 2007). The late spring/early summer period of the year in this area still has a considerable amount of sea ice, which limits the ability to a sample. Consequently, most of the studies focusing on the microbial eukaryotes in the Southern Ocean, including phytoplankton, were carried out during the January-April (summer/autumn) period when more open water and light availability have already produced the annual phytoplankton bloom (Flaviani et al., 2018; Clarke et al., 2019; Christaki et al., 2021; Lin et al., 2021). However, phytoplankton dynamics and environmental factors affecting them were examined by Arrigo et al. (2017) in early spring during the beginning of the bloom period. Light, and, consequently, the extent of sea ice seemed to be more important than nutrients in controlling phytoplankton production in early spring (Arrigo et al., 2017). Higher abundances of planktonic organisms were reported during the fall compared to the spring when the lowest abundance of nanoflagellates and bacteria was recorded (Clarke and Leakey, 1996; Ducklow et al., 2007; Trefault et al., 2021). Nevertheless, most of our environmental parameters, including inorganic nutrients, salinity, fluorescence, were in the same range as previously reported (Clarke and Leakey, 1996; Ducklow et al., 2013; Schofield et al., 2018; Christaki et al., 2021), especially prior to the phytoplankton bloom (Petrou et al., 2016; Arrigo et al., 2017; Trefault et al., 2021).

The mixed layer was shallower in our northern regions compared to southern regions (Factor 2; Table 1) comparable to previous observation (Schofield et al., 2018). Overall, the mixed layer was deeper than the chlorophyl maximum depth across the cruise, explaining the relative similarity between the samples taken at the sub-surface and at the DCM layer regarding both environmental parameters and planktonic community (Table 1 and Figures 1B,C). As noted above, reduced sea ice has been identified as a good predictor of net community production (Lin et al., 2021). The decrease in coverage and duration of sea ice observed prior to 2008 reversed temporarily and began increasing (Ducklow et al., 2013; Schofield et al., 2018). However, since 2015, the sea ice coverage has returned to its lower level of 2008, and the sea ice duration is continuing to decrease (see LTER data).^1^ This is expected to impact the productivity of the WAP system and could also affect community composition. A similar relationship has been observed between ice and heterotrophic protists in the Beauford Sea (Canadian Arctic), resulting in increased ammonium (Riedel et al., 2007). While a higher concentration of ammonium compared to previous studies was observed in the Southern Ocean as well (Clarke and Leakey, 1996; Christaki et al., 2021), this trend was not related to the ice coverage (Table 1). DIN, particularly nitrate/nitrate concentration, showed a latitudinal gradient and was negatively correlated to fluorescence and chlorophyll, suggesting a switch from new production supported by nitrite/nitrate in the northern regions to a more regenerated production in MB. However, we acknowledge that interpretation from a limited dataset (one cruise) can be risky, even if the correlation analysis is informative as a starting point (Supplementary Figure 1).

### Environment Differently Impacts the Diversity of the Pico-, Nano-, and Micro-Plankton

Overall, the diversity (richness and Chao1 index; Supplementary Figure 2A), assessed using 18S rRNA, was comparable to what was previously reported in other parts of the world ocean (de Vargas et al., 2015) and in the same region in other periods of the year (Lin et al., 2021). As noted previously, the spring corresponds to a low abundance of protists (Trefault et al., 2021), although, Arrigo et al. (2017) reported some areas of relatively high chlorophyll in early spring. Yet, even with the low abundance, each sample had an average of 1,000 OTUs (comparable to reports in other seasons; Lin et al., 2021), suggesting that most of these OTUs were active but in low abundance (a low signal in our community analysis based on RNA). This raises the question of the effect of environmental changes on this community.

Oxygen, salinity, temperature, and depth of the mixed layer are known factors playing a role in the eukaryotic richness (Raes et al., 2018), and, as mentioned before, sea ice and temperature are also good predictors of protist diversity in the WAP during the autumn (Lin et al., 2021), as they are, for this study, in late spring/early summer (Table 1 and Figure 1C). Lower diversity, in particular for the picoplankton, was observed in areas with less ice coverage, while a deeper mixed layer seemed to enhance the diversity of nanoplankton (Table 1). The difference in diversity in the north region compared to the south region of WAP has already been reported and linked with the depth of the mixed layer, the sea surface temperature, and the amount of sea ice (Schofield et al., 2018; Lin et al., 2021; Trefault et al., 2021), which mostly matches our own observations. It has also been noted that changes in productivity are related to the depth of the mixed layer (Montes-Hugo et al., 2009), with a shallower ZML, resulting in enhanced carbon fixation by phytoplankton (Schofield et al., 2018). In fact, increased sea ice correlated with an increase of photosynthetic efficiency because more micronutrients from the continental shelf were available, resulting in higher photosynthetic rates (Schofield et al., 2018). Low diversity in the Southern Ocean has been related to the bloom in summer months when phytoplankton biomass peaks and dissolved micronutrients tend to decrease (Arrigo et al., 2008; Ibarbalz et al., 2019). Based on these data, it is not unreasonable to expect that higher productivity associated with reduced sea ice will tend to decrease the protist diversity.

### Endemism in West Antarctic Peninsula

The late spring/early summer protistan community was dominated by the members of the SAR clade (Stramenopiles, Alveolates, and Rhizaria), with different compositions based on size fractions. Dominance of the eukaryotic SAR clade has been reported in almost all oceans (de Vargas et al., 2015; Pernice et al., 2015), including the Southern Ocean (Flaviani et al., 2018; Lin et al., 2021). Compared to a survey carried out in the world oceans (de Vargas et al., 2015), neither high proportion nor diversity of Foraminifera, Diplonemida or Collodaria was observed. Several possibilities exist that explain this observation. For example, it is strongly probable that Collodaria, which are mostly mesoplankton, were not captured in the Niskin bottles due to their large size (Biard et al., 2016). It is also possible that our primers did not amplify some lineages, such as Foraminifera, which has a limited diversity (Morard et al., 2018). Finally, these lineages may be rare, absent, not active, or seasonal in the Southern Ocean. Indeed, other surveys carried out in the Southern Ocean have not reported a large diversity or prevalence of Collodaria, Diplonemea, or Foraminifera (Sassenhagen et al., 2020; Lin et al., 2021).

The abundant and active taxa of late spring/early summer protistan community include *Corethron inerme* (also known as *Corethron criophilum var. inerme), Fragilariopsis kerguelensis, Porosira pseudodenticulata, Geminigera cryophila, and Phaeocystis antarctica*, which have been only described in Antarctica so far (Scott and Marchant, 2005). In addition to these phototrophic taxa, the ciliate *Cymatocylis calcyformis* has only been recently identified in the Southern Ocean (Kim et al., 2013). Other taxa have been described in similar environments such as *Micromonas polaris* in polar environments (Simon et al., 2017), or in different environments, such as the diatom parasites *Pirsonia formosa* and *P. guinardiae* in the North Sea (Kühn et al., 2004) and in WAP (Cleary and Durbin, 2016), the ciliate *Strombidium caudispina* in the South China Sea (Song et al., 2015), and the mixotrophic dinoflagellate *Heterocapsa rotundata* in the Northern hemisphere (Millette et al., 2015; Guiry and Guiry, 2018). Something to consider here too is that the taxonomic assignment of dinoflagellates using a short fragment of the SSU ribosomal DNA (or 18S rRNA) is difficult, given the lack of variability, resulting in multiple taxa matching the same OTU at the same level of similarity. For example, our taxonomic analysis by similarity concludes that most of the OTUs related to dinoflagellates were closely related to *Heterocapsa rotundata* KY980129 or *(1) Gyrodinium cf. spirale* KP790157, *or (2) Prorocentrum sp.* MN824022, without a clear cutoff. In the same way, we can also identify OTUs matching *Symbiodinium* by similarity but *Gyrodinium spirale, Gymnodinium* sp. or unidentified dinoflagellates by phylogeny (Supplementary Figure 3B).

### Community and Size Fractions

The use of three size fractions allows us to look at community structure at a finer scale. Overall, each size fraction is impacted differently by environmental factors. For example, picoplankton distribution has a stronger association with salinity compared to nano- and micro-plankton, while oxygen showed a slightly stronger impact on micro- and nano-plankton (Figure 1 and Supplementary Figure 1). The dominance of diatoms and dinoflagellates has been reported in the Ross Sea during the austral summer and in WAP during the fall (Wolf et al., 2013; Lin et al., 2021) and early spring (Arrigo et al., 2017). The diatoms were dominant but mostly within the microplankton (>20 μm), while dinoflagellates were abundant in the pico- and nano-size fractions (0.2–5 and 5–20 μm, respectively). As, in January of 2012–2016 (Lin et al., 2021), our dinoflagellate community was dominated by the genus *Gyrodinium*. However, the diatom community is mostly different as our microplanktonic community was dominated by the large diatom *Corethron inerme*, while the phytoplankton community was dominated by *Fragilariopsis, Chaetoceros*, and *Proboscia* in January–February 2014 (Lin et al., 2017), and by *Thalassiosira*, *Odontella*, *Porosira*, *Actinocyclus*, *Proboscia*, and *Chaetoceros* in January 2012–2016 (Lin et al., 2021), or *Eucampia* in the Amundsen Sea in 2010 (Wolf et al., 2013). In early spring, Arrigo et al. (2017) found *Phaeocystis antarctica* and diatoms to consistently exceed 90% of the phytoplankton biomass in pigment and microscopic analyses, with *P. antarctica* and diatoms positively and negatively correlated, respectively, with sea ice. Cryptophytes and chlorophytes were occasionally significant components in their study, but dinoflagellates never contributed more than a few percent to the phytoplankton community (Arrigo et al., 2017).

Large diatoms are known consumers of nitrate, especially during the early summer (Clarke and Leakey, 1996), and the concentration of DIN (both NH_4_^+^ and NO_3_^–^/NO_2_^–^) was relatively high during the study period, which was also marked by a high level of light. Some diatoms (e.g., *Corethron*) are adapted to higher UV intensity by downregulating protein to protect their photosystem II, mitigating the impact of photosynthetic inhibition (Read et al., 2019). This can explain the inverse relationship between PAR and the concentration of dissolved oxygen or oxygen saturation observed due to a reduced photosynthesis rate (Supplementary Table 1 and Figure 1). Dominance of large diatoms can be related to shallow mixed layer water masses in the north regions, while nanoflagellates are more abundant in a deeper mixed layer zone because of their photoadaptation (Sakshaug and Holm-Hansen, 1986; Villafañe et al., 1995). In our study, the mixed layer depths were deeper in the MB and OFF groups, where higher importance of picophytoplankton, such as *Micromonas* and *Pelagomonas*, and nanoplanktonic diatoms, such as *Chaetoceros* (Figures 1, 2), was observed. In addition, *Pelagomonas* can frequently dominate nitrate assimilation in other environments (Dupont et al., 2015) as this taxon relies on nitrate as the primary source of N (Choi et al., 2020) and has been reported as an important player in oligotrophic environments.

### Heterotrophy and Diversity

Heterotrophic protists, including dinoflagellates, have been related to higher concentrations of ammonium, mostly due to their role in regenerating NH_4_^+^ (Glibert, 1993, 1998) and the key role of NH_4_^+^ in the ice environments (Riedel et al., 2007). Heterotrophic protists, in particular ciliates, showed a positive relationship with the amount of ice in the Amundsen Sea (Wolf et al., 2013), while the opposite trend was observed here (Supplementary Figure 1B). Ciliates are considered to have an important role in the consumption of pico-nanoplankton during night migration in summer along the WAP (Alcamán-Arias et al., 2018). In our data, an inverse relation between ciliates and cryptophytes was observed, which can suggest a predator-prey relationship (Figure 2B). However, the presence/dominance of cryptophytes in low salinity and warm stratified water in WAP was previously observed and related to their preferential growth within the surface of melting water (Ferreira et al., 2020; Lin et al., 2021), which also may explain the inverse relationship observed between ice coverage and ciliates. However, the collinearity within our measured environmental parameters, mostly related to the latitudinal gradient, complicates the assessment of the impact of each environmental parameter on the community distribution (Supplementary Table 2). Nevertheless, permutational multivariate ANOVA using distance matrices (adonis2), and an Akaike information criterion corrected showed that the pico- and nano-plankton are mostly impacted by latitude (21%) and NO_2_NO_3_ (12%), while the microplankton is also shaped by the latitude (23%), the depth of the photic zone (11%), and the number of particles in the water (7%; beam transmission; Supplementary Table 3) played a role in the distribution of the microplanktonic community. However, additional data are needed to confirm this observation and disentangle the effect of the latitudinal gradient to the effect of each environmental parameter.

The overall diversity, based on 18S rRNA (Supplementary Figure 2), tends to increase toward the south, which also corresponded to an increase of heterotrophy, including both phagotrophy and mixotrophy. Overall, the ratio autotroph/heterotroph decreases with an increasing latitude toward the south. Most of this change is related to an increase in the kleptoplastidic ciliates, heterotroph dinoflagellates, and parasitic Stramenopiles, while mixotrophic cryptophytes decrease (Figure 2). Garzio and Steinberg (2013) showed an increase of microzooplankton (heterotrophic protists) abundance and biomass toward the south in WAP, with higher values observed in the MB area. The Marguerite Bay is a known biodiversity hotspot due to currents bringing warmer water and nutrients from the deep water (Martinson et al., 2008; Ducklow et al., 2012), resulting in high primary production (Clarke et al., 2008; Vernet et al., 2008) and high populations of krill and penguins (Ashjian et al., 2004; Friedlaender et al., 2011). The higher proportion of heterotrophs may have led to a more complex and diversified community and, therefore, to higher diversity.

## Conclusion

The results of the current study show a strong latitudinal gradient in protistan diversity and activity that is in agreement with other studies, describing phytoplankton and microzooplankton, mostly later in the spring (Garzio and Steinberg, 2013; Arrigo et al., 2017; Lin et al., 2021). Overall, the Marguerite Bay area showed the most distinctive environmental parameters, the most distinct pico-, nano-, and micro-planktonic communities, and the highest number of specific OTUs, suggesting that the environment played an important role in shaping the community in the WAP. Although these differences in diversity and activity were relatively small, so were the environmental changes, suggesting that the climate change, either directly (temperature) or indirectly, could have a significant effect on the microbial eukaryotic community for which we gathered these initial data.

## Data Availability Statement

The datasets presented in this study can be found in online repositories. The names of the repository/repositories and accession number(s) can be found below: NCBI – PRJNA807326. Supplementary Datasets 1–3 can be found here: doi: 10.6084/m9.figshare.19514110.v3.

## Author Contributions

J-DG, WJ, and RS participated in the cruise, and with RG conceived and designed the experiments. J-DG performed the RNA community experiments and analyzed the data. WJ performed the nutrients and production analyses. J-DG wrote the manuscript with contribution from all the authors. All authors read and approved the final manuscript.

## Conflict of Interest

The authors declare that the research was conducted in the absence of any commercial or financial relationships that could be construed as a potential conflict of interest.

## Publisher’s Note

All claims expressed in this article are solely those of the authors and do not necessarily represent those of their affiliated organizations, or those of the publisher, the editors and the reviewers. Any product that may be evaluated in this article, or claim that may be made by its manufacturer, is not guaranteed or endorsed by the publisher.

## Footnotes

1
http://pal.lternet.edu/data

## Supplementary Material

Supplementary Dataset 1 The OTU table using paired-end reads. T1-5 are the taxonomic ranks, acc the GenBank accession number of the corresponding reference, id% is the percentage of identity, occurrence represents the number of samples in which the OTUs were present, read number the total read number. The samples are labeled as following: station_depthlayer_size._20 for the microsize, _5 for the nanosize, and _0.2 for the picosize.

Supplementary Dataset 2 The OTU table using forward reads. Accession is the GenBank accession number of the corresponding reference; Btaxo_rank1-6, Bgenus, and Bsp are the taxonomic ranks from PR2, occurrence represents the number of samples in which the OTUs was present, read number the total read number. The samples are labeled as the following: station_depthlayer_size. _20 for the microsize, _5 for the nanosize, and _0.2 for the picosize.

Supplementary Dataset 3 The OTU table using paired-end reads with at least 10 reads and then rarefied. T1-5 are the taxonomic ranks, acc the GenBank accession number of the corresponding reference, id% is the percentage of identity, Trophic is the trophic mode for the OTUs based on the literature. Reference_Trophic is the reference if different from the main text, occurrence represents the number of the samples in which the OTUs were present, read number the total read number. The samples are labeled as the following: station_depthlayer_size; _20 for the microsize, _5 for the nanosize, and _0.2 for the picosize.
