# Supplementary material for: Diversity of Microbial Eukaryotes Along the West Antarctic Peninsula in Austral Spring
Source: Front Microbiol. 2022 May 16;13:844856. doi: 10.3389/fmicb.2022.844856 (PMC9149413; doi:10.3389/fmicb.2022.844856)
Supplement: Supplementary Dataset 1 — The OTU table using paired-end reads. T1-5 are the taxonomic ranks, acc the GenBank accession number of the corresponding reference, id% is the percentage of identity, occurrence represents the number of samples in which the OTUs were present, read number the total read number. The samples are labeled as following: station_depthlayer_size._20 for the microsize, _5 for the nanosize, and _0.2 for the picosize. [file Data_Sheet_1.docx]

**Diversity of microbial eukaryotes along the West Antarctic peninsula in austral spring**

Jean-David Grattepanche^1*^, Wade H Jeffrey^2^, Rebecca J Gast^3^, Robert W Sanders^1^

**Table S1.** Statistical analysis of the NMDS using (A) envfit; and (B) Adonis (permutational multivariate analysis of variance using distance matrices) to disentangle the effect of environmental parameters

A- NMDS and envfit analysis

|  | **Picoplankton (0.2-5µm)** | | | | | **Nanoplankton (5-20µm)** | | | | | | **Microplankton (>20µm)** | | | | | |  |
| --- | --- | --- | --- | --- | --- | --- | --- | --- | --- | --- | --- | --- | --- | --- | --- | --- | --- | --- |
|  | NMDS1 | NMDS2 | r2 | Pr(>r) |  | | NMDS1 | NMDS2 | r2 | Pr(>r) |  | | NMDS1 | NMDS2 | r2 | Pr(>r) |  | |
| time | 0.02259 | -0.99974 | 0.3548 | 0.002 | ** | | -0.69611 | 0.71794 | 0.1865 | 0.053 | . | | 0.8403 | -0.54212 | 0.2789 | 0.013 | * | |
| latitude | 0.83357 | 0.55241 | 0.8009 | 0.001 | *** | | -0.61872 | -0.78561 | 0.7592 | 0.001 | *** | | -0.88347 | -0.46849 | 0.82 | 0.001 | *** | |
| longitude | 0.80867 | 0.58826 | 0.6196 | 0.001 | *** | | -0.67009 | -0.74228 | 0.4511 | 0.001 | *** | | -0.801 | -0.59866 | 0.5667 | 0.001 | *** | |
| Ice Coverage | 0.95171 | -0.30699 | 0.0592 | 0.418 |  | | -0.48902 | -0.87227 | 0.6407 | 0.001 | *** | | -0.91514 | 0.40313 | 0.2001 | 0.038 | * | |
| depth (m) | -0.31693 | 0.94845 | 0.4502 | 0.001 | *** | | 0.97878 | -0.20492 | 0.2479 | 0.016 | * | | -0.113 | 0.9936 | 0.0944 | 0.229 |  | |
| PAR | 0.96756 | 0.25263 | 0.1934 | 0.039 | * | | -0.93516 | -0.35423 | 0.106 | 0.221 |  | | -0.36686 | -0.93028 | 0.1211 | 0.157 |  | |
| air temperature | 0.90958 | 0.41554 | 0.2765 | 0.004 | ** | | -0.79652 | -0.60461 | 0.1356 | 0.152 |  | | -0.91576 | -0.40172 | 0.0831 | 0.289 |  | |
| water temperature | 0.87448 | 0.48506 | 0.3534 | 0.003 | ** | | -0.8572 | 0.51499 | 0.1277 | 0.161 |  | | -0.12489 | -0.99217 | 0.2169 | 0.027 | * | |
| salinity | 0.13663 | 0.99062 | 0.6798 | 0.001 | *** | | 0.53115 | -0.84728 | 0.2265 | 0.031 | * | | -0.7257 | 0.68801 | 0.2215 | 0.03 | * | |
| oxygen | -0.14826 | -0.98895 | 0.4144 | 0.001 | *** | | -0.09677 | 0.99531 | 0.4886 | 0.001 | *** | | 0.67164 | -0.74088 | 0.5015 | 0.001 | *** | |
| beam transmission | -0.50366 | 0.8639 | 0.2772 | 0.005 | ** | | 0.62312 | -0.78213 | 0.3112 | 0.013 | * | | -0.31418 | 0.94936 | 0.4334 | 0.001 | *** | |
| fluorescence | -0.21458 | -0.97671 | 0.137 | 0.111 |  | | -0.08788 | 0.99613 | 0.3727 | 0.003 | ** | | 0.62452 | -0.78101 | 0.4984 | 0.001 | *** | |

Note: Significance: 0 ‘***’ 0.001 ‘**’ 0.01 ‘*’ 0.05 ‘.’ 0.1 ‘ ’ 1; Permutation: 999 free

B- Adonis analysis for whole community

| **Terms added sequentially**  **(first to last)** | **Df** | **Sums Of Sqs** | **Mean Sqs** | **F.Model** | **R^2^** | **Pr (>F)** |  |
| --- | --- | --- | --- | --- | --- | --- | --- |
| size | 2 | 8.556 | 4.2782 | 18.1823 | 0.22366 | 0.0001 | *** |
| Group | 3 | 3.674 | 1.2248 | 5.2055 | 0.09605 | 0.0001 | *** |
| latitude | 1 | 0.638 | 0.638 | 2.7114 | 0.01668 | 0.0007 | *** |
| layer | 2 | 0.767 | 0.3837 | 1.6306 | 0.02006 | 0.0123 | * |
| size:Group | 6 | 4.799 | 0.7999 | 3.3994 | 0.12545 | 0.0001 | *** |
| size:latitude | 2 | 1.006 | 0.503 | 2.1375 | 0.02629 | 0.0007 | *** |
| Group:latitude | 3 | 1.185 | 0.395 | 1.6786 | 0.03097 | 0.0035 | ** |
| size:layer | 4 | 1.063 | 0.2657 | 1.1294 | 0.02778 | 0.1881 |  |
| Group:layer | 4 | 0.91 | 0.2275 | 0.9669 | 0.02379 | 0.5359 |  |
| latitude:layer | 1 | 0.2 | 0.1998 | 0.8492 | 0.00522 | 0.6563 |  |
| size:Group:latitude | 5 | 1.734 | 0.3468 | 1.474 | 0.04533 | 0.0034 | ** |
| size:Group:layer | 8 | 1.505 | 0.1881 | 0.7996 | 0.03934 | 0.9834 |  |
| size:latitude:layer | 2 | 0.341 | 0.1706 | 0.7249 | 0.00892 | 0.94 |  |
| Group:latitude:layer | 3 | 0.573 | 0.1909 | 0.8114 | 0.01497 | 0.8781 |  |
| size:Group:latitude:layer | 5 | 0.951 | 0.1902 | 0.8085 | 0.02486 | 0.9435 |  |
| **Residuals** | 44 | 10.353 | 0.2353 |  | 0.27062 |  |  |
| **Total** | 95 | 38.256 |  |  | 1 |  |  |

**Table S2: Collinearity and correlation analyses**: the strong latitudinal gradient complicates the estimate of the effect of each environmental parameter on the community distribution. VIF= Variance inflation factor; R2 represent the correlation value and P the associated probability (also shown in Figure S1 A).

| Variables | Tolerance | VIF | R2 | P |  |
| --- | --- | --- | --- | --- | --- |
| group | 0.001 | 816.07 | -0.90 | 0.000 | *** |
| ZML_TS | 0.009 | 107.38 | -0.76 | 0.000 | *** |
| oxygen | 0.009 | 115.44 | -0.47 | 0.000 | *** |
| O_saturation | 0.000 | 294675.10 | -0.42 | 0.000 | *** |
| fluorescence | 0.008 | 126.46 | -0.30 | 0.003 | * |
| time | 0.043 | 23.10 | -0.18 | 0.080 | . |
| ZCM | 0.019 | 52.77 | -0.15 | 0.100 | . |
| beam_trans | 0.001 | 1121.81 | -0.09 | 0.400 |  |
| depth | 0.003 | 398.10 | -0.06 | 0.600 |  |
| Bprod | 0.096 | 10.41 | 0.01 | 0.900 |  |
| Ze | 0.010 | 102.13 | 0.07 | 0.500 |  |
| salinity | 0.000 | 207517.30 | 0.29 | 0.004 | * |
| NH4 | 0.010 | 98.05 | 0.35 | 0.001 | *** |
| bottom | 0.033 | 29.95 | 0.35 | 0.000 | *** |
| PAR | 0.128 | 7.79 | 0.36 | 0.000 | *** |
| water_temp | 0.000 | 3048045.00 | 0.40 | 0.000 | *** |
| conductivity | 0.000 | 2439110.00 | 0.43 | 0.000 | *** |
| surf_PAR | 0.004 | 269.09 | 0.44 | 0.000 | *** |
| air_temp | 0.023 | 43.02 | 0.45 | 0.000 | *** |
| PO4 | 0.191 | 5.23 | 0.47 | 0.000 | *** |
| ice | 0.014 | 73.03 | 0.48 | 0.000 | *** |
| NO2NO3 | **0.011** | **93.59** | **0.67** | **0.000** | ******* |
| longitude | 0.002 | 405.56 | 0.87 | 0.000 | *** |

Note: Collinearity are show in red (VIF > 20 and Tolerance < 0.1),

Significance: 0 ‘***’ 0.001 ‘**’ 0.01 ‘*’ 0.05 ‘.’ 0.1 ‘ ’ 1

**Table S3: summary of Adonis2 results for each size fraction**. Environmental parameters for the model are selected based on Akaike information criterion corrected.

1. Picoplankton

|  | Df | SumOfSqs | R2 | F | Pr(>F) |  |
| --- | --- | --- | --- | --- | --- | --- |
| latitude | 1 | 1.6428 | 0.21239 | 7.9624 | 1.00E-04 | *** |
| NO2NO3 | 1 | 0.934 | 0.12075 | 4.5269 | 3.00E-04 | *** |
| Residual | 25 | 5.1579 | 0.66686 |  |  |  |
| Total | 27 | 7.7347 | 1 |  |  |  |

1. Nanoplankton

|  | Df | SumOfSqs | R2 | F | Pr(>F) |  |
| --- | --- | --- | --- | --- | --- | --- |
| latitude | 1 | 1.317 | 0.20955 | 6.9091 | 1.00E-04 | *** |
| NO2NO3 | 1 | 0.7743 | 0.12321 | 4.0623 | 2.00E-04 | *** |
| Residual | 22 | 4.1935 | 0.66724 |  |  |  |
| Total | 24 | 6.2849 | 1 |  |  |  |

1. Microplankton

|  | Df | SumOfSqs | R2 | F | Pr(>F) |  |
| --- | --- | --- | --- | --- | --- | --- |
| latitude | 1 | 2.0129 | 0.2359 | 9.6708 | 0.0001 | *** |
| Ze | 1 | 0.9155 | 0.10729 | 4.3985 | 0.0001 | *** |
| beam_trans | 1 | 0.6091 | 0.07138 | 2.9264 | 0.0027 | ** |
| Residual | 24 | 4.9955 | 0.58543 |  |  |  |
| Total | 27 | 8.5331 | 1 |  |  |  |

Note: Significance: 0 ‘***’ 0.001 ‘**’ 0.01 ‘*’ 0.05 ‘.’ 0.1 ‘ ’ 1

**Figure S1.** Correlation between (A) environmental parameters (including alpha diversity), (B) between lineages (class) and environmental parameters.

| A |  |
| --- | --- |
| B |  |

**Figure S2.** Alpha-diversity (Richness, Shannon and Chao1 indices) considering (A) OTUs and (B) morphospecies for each sample separated by size fractions and depth layers is slightly higher for the samples collected in Marguerite Bay (southern samples, dark blue).

A-

B-


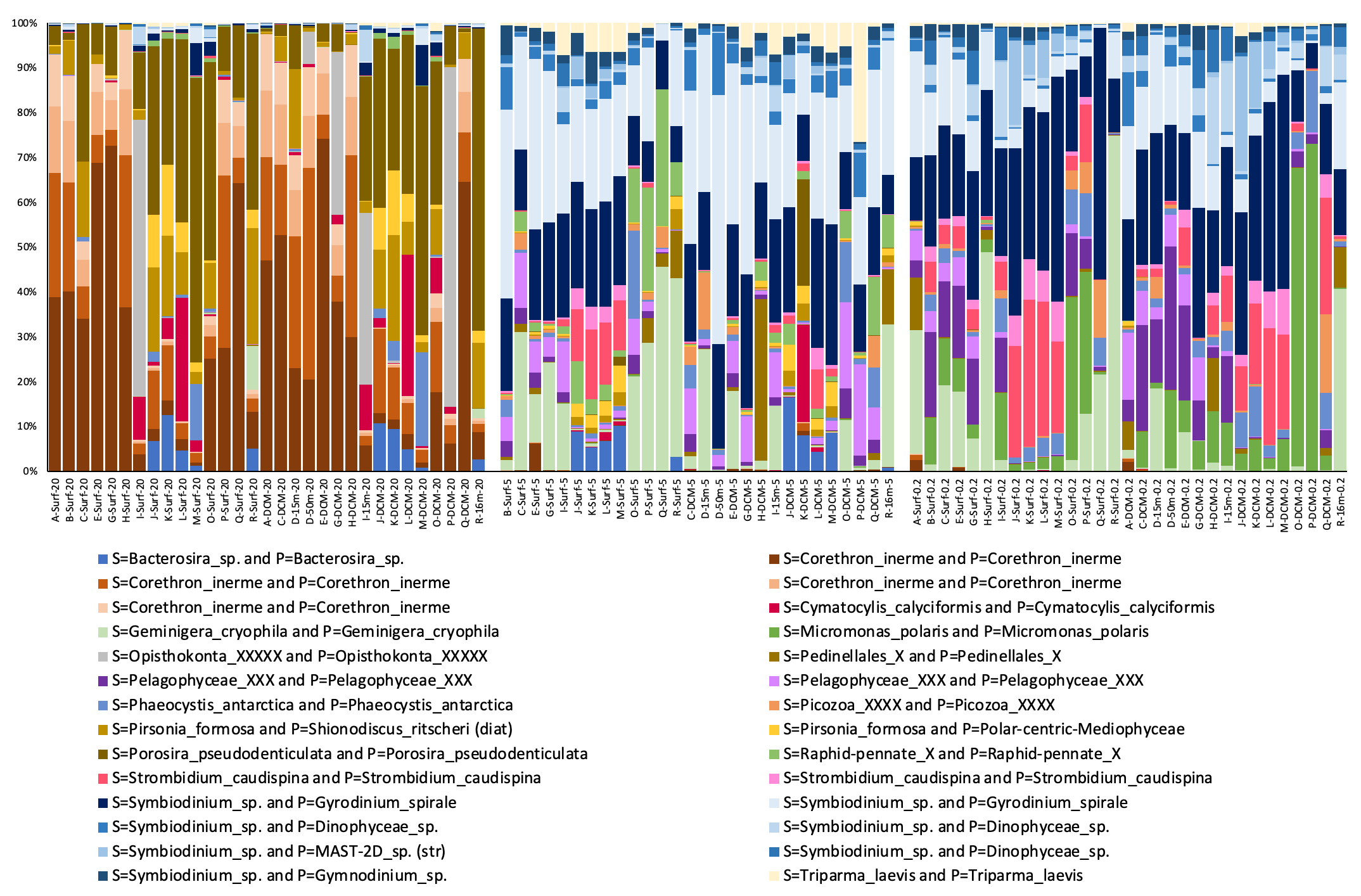


**Figure S3**. Distribution of (A) main lineages and (B) OTUs representing at least 1% of the total reads number. Each OTUs is assigned to the closest morphospecies. Here again, we can clearly observe the distinct composition of micro-, nano- and picoplankton. For the bottom bar graph, S= refer to assignment per similarity and P= to assignment by phylogeny.


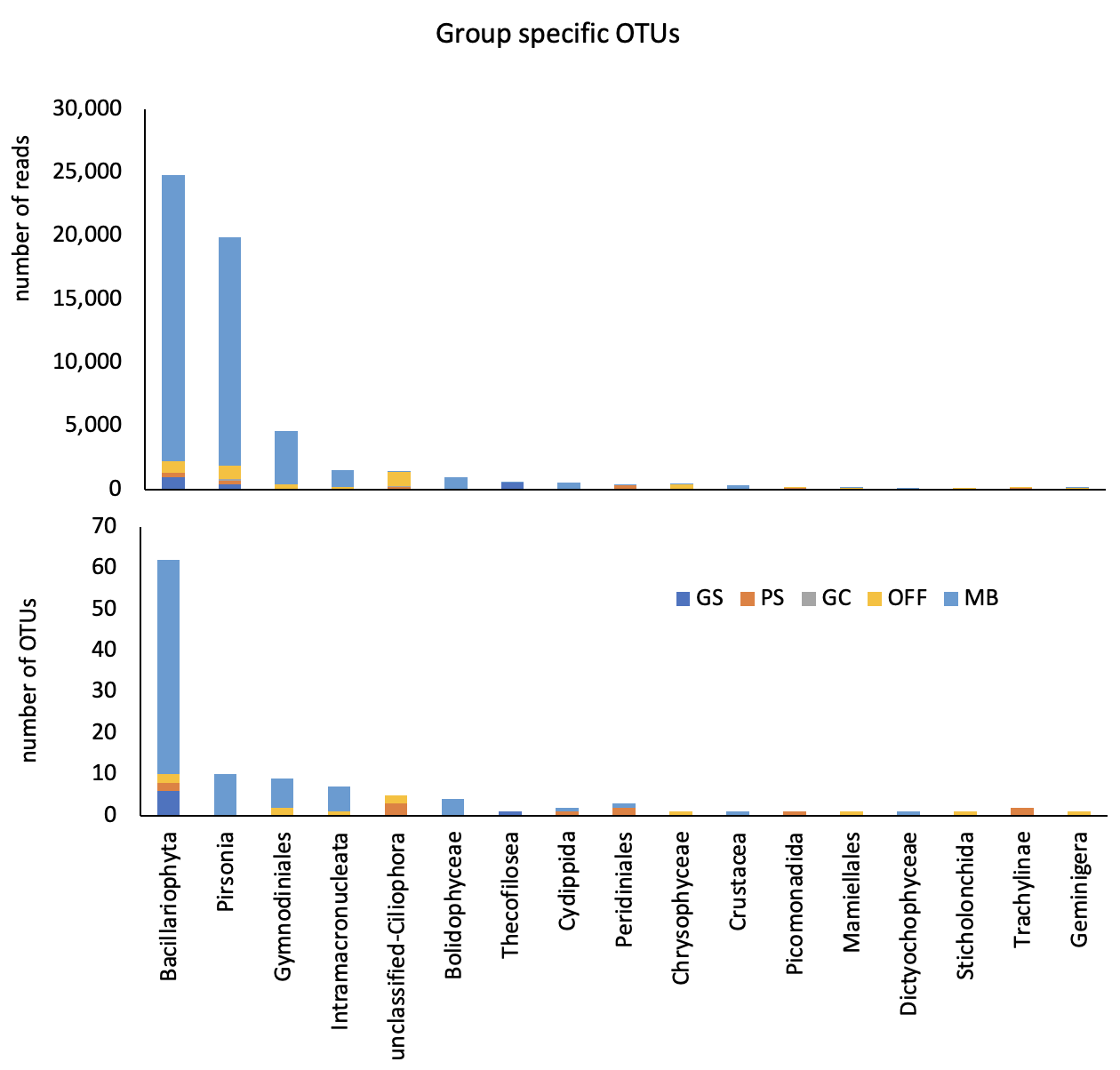


**Figure S4**. Distribution of OTUs specific to each group. Each OTUs is assigned to the closest morphospecies. OTUs are assigned to an environmental group if 90% of its read are observed in this group.
